# Adaptation and Validation of the Preparation for Future Care Needs Scale for Chinese Older Adults in Hong Kong

**DOI:** 10.1093/geront/gnab089

**Published:** 2021-06-24

**Authors:** Xue Bai, Chang Liu, Yajun Song, Silvia Sörensen

**Affiliations:** Department of Applied Social Sciences, The Hong Kong Polytechnic University, China; Institute of Active Ageing, The Hong Kong Polytechnic University, China; Department of Applied Social Sciences, The Hong Kong Polytechnic University, China; School of Social and Public Administration, East China University of Science and Technology, Shanghai, China; Warner School of Education and Human Development, University of Rochester, New York, USA

**Keywords:** Aging adults, Care planning, Proactive coping, Scale validation

## Abstract

**Background and Objectives:**

Care planning can protect against or offset potential stressors in the caregiving stage and mitigate their detrimental effects. This study aimed to translate, adapt, and validate 2 short forms of the multidimensional, theory-guided scale measuring preparation for future care needs (PFCN) among Chinese older adults in Hong Kong.

**Research Design and Methods:**

Data were derived from a cross-sectional survey of 862 community-dwelling individuals aged 60 years and older. Exploratory factor analysis (EFA) and confirmatory factor analysis (CFA) were conducted to assess the structural validity of the scales. Criterion-related validity, known-groups validity, and internal consistency were also examined.

**Results:**

EFA yielded a 14-item 4-factor (awareness, avoidance, decision making, and concrete planning) model, which was supported by CFA and explained 68.9% of the total variance. CFA also supported the structural validity of the 5-item scale. Criterion-related validity of the 2 scales was supported by their significant and positive correlations with domain-specific planning behaviors for retirement. Known-groups validity of the 2 scales was demonstrated by significant differences in scores between male and female older adults and scores between different educational levels and socioeconomic status. Cronbach’s alphas for the internal consistency of the 14-item and 5-item scales were 0.889 and 0.774, respectively.

**Discussion and Implications:**

PFCN scales enable researchers and service practitioners to accurately understand and assess older adults’ processes and efforts in care planning, facilitate the identification of individuals at risk from inadequate planning, and inform the development of interventions to enhance care preparation in target domains.

The increasing need for care among older people caused by deteriorating health is a potential stressor affecting older adults, their families, and society. According to proactive coping theory (Aspinwall & Taylor, 1997), care planning before intensive care represents a form of proactive coping to prevent or offset potential stressors and mitigate their detrimental effects. Empirical studies have revealed that effective care planning is related to less insecurity about the future (Sörensen & Pinquart, 2000a), reduced depression and anxiety in older adults (Sörensen et al., 2012), and improved concordance between older adults’ care preferences and delivered care (Brinkman-Stoppelenburg et al., 2014; Houben et al., 2014). Meanwhile, care planning can also benefit potential caregivers by guiding decisions during crises (Pinquart & Sörensen, 2002; Sörensen et al., 2011).

Scholars have generally conceptualized care planning in terms of the detailed content of care plans and preparation processes. With respect to care plan content, studies have examined older adults’ future care plans with an emphasis on residence location, expected caregivers and services, and domain-specific preparation for old age (He & Chou, 2019; Kornadt et al., 2019; Lum et al., 2016). Sörensen and colleagues (Sörensen & Pinquart, 2001; Sörensen et al., 2017) conceptualized care planning as a dynamic process that involves attitudinal and behavioral components such as awareness or avoidance of future care needs, gathering information, decision making, and concrete planning. This model has been used in diverse community- and institution-dwelling aging populations in North America, Europe, and mainland China (Pinquart et al., 2003; Song et al., 2018; Sörensen & Pinquart, 2001; Sörensen et al., 2017).

The conceptualization of the care planning process has been based on several psychological and health behavior theories (Sörensen & Pinquart, 2001; Sörensen et al., 2017). According to cognitive theories of planning (e.g., Berg et al., 1997; Scholnick & Friedman, 1993), this process generally includes raising awareness of the need to plan, gathering information, determining goals and assessing options to achieve them, choosing specific options, implementing plans, and evaluating their effectiveness (Sörensen et al., 2017). Concerning the process of translating awareness to active planning behaviors, the revised protection motivation theory (Maddux & Rogers, 1983) suggests that a threat appraisal of the severity of an event, high probability of event occurrence, high efficacy of a recommended coping response, and high self-efficacy expectancy will activate planning activities. However, threat appraisals emphasizing the severity of care needs and perceived vulnerability could inhibit older adults from taking further action (Aspinwall & Taylor, 1997; Sörensen et al., 2017).

Following the abovementioned cognitive process of planning, Sörensen and Pinquart (2001) developed a 29-item scale to assess the process of preparation for future care needs (PFCN). In an attempt to promote the wider and easier implementation of the scale in both academic and clinical settings, two short forms, namely the 15-item and 5-item PFCN scales, were introduced (Sörensen et al., 2017). Questions on the three versions of the PFCN scale cover five critical domains: (a) awareness of future care needs (e.g., “I pay close attention to how my physical and mental capabilities are changing to assess whether I may soon need help or care”), (b) gathering information about future care (e.g., “I have gathered information about options for care by talking to friends and/or relatives”), (c) making decisions about one’s care preference (e.g., “I know what options for care I don’t want”), (d) concrete planning activities including communication of care preferences with family members and plan initiation (e.g., “I have explained to someone close to me what my care preferences are”), and (e) avoidance of care planning (e.g., “I try not to think about things like future loss of independence”) (Sörensen et al., 2017).

Awareness, gathering information, decision making, and concrete planning domains were reported to be positively correlated, and the avoidance domain had a slightly positive correlation with the awareness domain and a negative correlation with the concrete planning domain (Sörensen et al., 2017). Moreover, early steps in the care planning process may predict later ones (Song et al., 2018; Sörensen & Pinquart, 2000b). Studies have further found that older women are more likely to have awareness of their long-term care needs than men are (He & Chou, 2019). Those with higher educational attainment and socioeconomic status are more likely to engage in care planning, especially awareness and gathering information (Kawakami et al., 2021; Song, 2016).

Like many other developed societies, Hong Kong has a rapidly aging population and faces a growing need for eldercare. More than 70% of community-dwelling older adults in Hong Kong are estimated to have chronic diseases, and approximately 25% require assistance with daily living activities (Census and Statistics Department, 2009). Recent cases of violence in informal caregiving situations in Hong Kong (Blundy, 2017; Kao, 2017) have renewed calls for effective planning for future care needs in families with older adults. Studies of older adults in Hong Kong have examined intergenerational relationships, care plans, and diverse care expectation patterns (Bai, 2018, 2019a, 2019b; Bai et al., 2020; Kornadt et al., 2019). However, the dynamics of their planning process remains unclear. A validated measurement that assesses the preparatory process of Chinese older adults regarding their future care needs may address these research gaps and help identify opportunities for intervention and support during the process.

It is not entirely clear whether the original PFCN scales are suitable for assessing care planning behaviors of older adults in Hong Kong because of the potential differences in the sociocultural contexts of eldercare in Hong Kong and Western countries. For instance, the taboo on thinking and talking about death is a powerful cultural barrier to care planning in Chinese societies (Yap et al., 2018). Insofar as older people’s care needs are a death-related topic, older Chinese people may avoid discussing future care plans with their family members or be reluctant to engage in certain concrete planning activities. Meanwhile, the traditional Confucian value of filial piety places a default expectation on adult children to care for older people in Asian Chinese communities, which results in the underdevelopment of formal care policies and services (Chow, 2006). These sociocultural factors may influence Chinese older adults’ interest in care planning and lead to differing planning patterns. Therefore, this study investigated the suitability of the 15-item and 5-item versions of the PFCN scale and then translated, adapted, and validated the items to assess the care planning process of Chinese older adults in Hong Kong.

The factorial structure, criterion-related validity, known-groups validity, and internal consistency of the scale were tested. To examine the criterion-related validity of the scale, we hypothesized that the level of care planning would be positively correlated with domain-specific retirement planning behaviors because the scales related to retirement planning were also intended to capture goal-oriented thoughts and behaviors. Meanwhile, based on the abovementioned empirical evidence, we hypothesized that known-groups validity would be supported by higher levels of care planning among older women, and older adults of higher educational levels and socioeconomic status.

## Research Method

### Participants and Data Collection

A face-to-face questionnaire survey was conducted from November 2017 to May 2018. Details of the sampling strategy and data collection methods are available in another paper (Liu et al., 2021). Data of 862 participants aged 60 years or older were extracted for use in the current study.

### Measurement

#### PFCN-14

Several steps were taken to adapt the original PFCN scale and to ensure the scale was culturally appropriate for measuring care planning in Chinese older adults, including evaluation of content validity by an expert panel, item revision, and forward and backward translation.

An expert panel consisting of five scholars with expertise in social work, social policy, social gerontology, sociology, and psychology was involved in the study. The experts reviewed and evaluated whether each item was relevant, crucial, and clear to measure care planning process in Hong Kong older adults. Meanwhile, 21 individual interviews were conducted to collect Hong Kong older adults’ views about care planning and examine whether their care planning process could be captured by the original PFCN-15 or if revisions were required. The results revealed that Hong Kong older adults’ care planning behaviors were largely similar to the Western behaviors described by Sörensen and Pinquart (2000a; Pinquart & Sörensen, 2002). One prominent difference is that Chinese older adults are likely to rely on family members to collect information or even make decisions about future care arrangements. Other differences are that they often compare care options on the basis of personal preference rather than relevant information and tend to gather information passively rather than proactively. However, these findings did not motivate the expert panel to make major revisions to the scale because the general “process” of planning among Chinese older adults was consistent with that of their Western counterparts.

Concerning the usability of the items in the original scale, both the results of individual interviews and the evaluation by the expert panel indicated that most items were applicable to the Hong Kong context, except that no participants brought up notions of “writing down” or “making a record of” their care preferences (Item 14: “I have written down my preferences for care”). They generally expressed a negative or evasive attitude toward such actions. In Chinese culture, writing down care preferences may be perceived as morbid and too closely related to death. Such expression may cause discomfort and be considered a source of misfortune for Chinese older adults, thus causing them to avoid writing their preferences.

The original English version of the 15-item PFCN scale (Sörensen et al., 2017) was translated into Chinese by a bilingual scholar and then reverse-translated by another scholar. The two English versions were examined by two scholars, who observed no major discrepancies. The 15-item Chinese version was then used in a questionnaire survey with 166 Hong Kong older adults to ensure that the instrument would be clearly understood by respondents. In the results, Item 14 (“I have written down my preferences for care”) yielded a relatively low mean score of 1.097 (score range 1–5) and a standard deviation (*SD*) of 0.497. The mean scores of other items ranged from 1.394 to 3.503, with *SD*s near or higher than 1.00. This result suggested that the responses to Item 14 were concentrated at the lower end of the scale and the item’s variability was considerably lower than the suggested threshold (≥1.00; Gamst et al., 2015). In addition, the item was the only one with a corrected item-total correlation (i.e., the correlation between each item and the total scale score that excludes that item; *r* = 0.182) lower than the recommended level of 0.20 (Streiner & Norman, 2003), indicating a weak correlation with other scale items. Thus, on the basis of the qualitative and quantitative evidence, the expert panel reached the consensus that the item was culturally inappropriate and decided to remove it. No revision was made to this item, and no item was added to replace it because no relevant alternative activities were mentioned by participants during interviews, and two existing items measured the same domain of care planning.

The modified 14-item Chinese version of the PFCN scale was used to measure aging adults’ care preparation processes. The scale measured five subdomains, namely awareness, avoidance, gathering information, decision making, and concrete planning. The participants rated each item on a 5-point scale from 1 (*not at all true*) to 5 (*completely true*). The sum score was calculated by adding together the scores of single items. Total scores ranged from 14 to 70, with a higher score indicating more adequate care preparation. Cronbach’s alpha, representing internal consistency, was 0.889 for the current sample.

#### PFCN-5

The 5-item version of the PFCN scale consists of items extracted from five domains of the PFCN-15 scale (Sörensen et al., 2017). Specifically, Item 3 (“Talking to other people has made me think about whether I might need help or care in the future”) was extracted from the awareness domain, Item 4 (“I try not to think about things like future loss of independence”) was extracted from the avoidance domain, Item 8 (“I have gathered information about options for care by talking to friends and/or relatives”) was extracted from the gathering information domain, Item 12 (“If I ever need help or care, I can choose between several options that I have considered in some depth”) was extracted from the decision-making domain, and Item 13 (“I have explained to someone close to me what my care preferences are”) was extracted from the concrete planning domain. Total scores ranged from 5 to 25. Cronbach’s alpha was 0.774 for the current sample.

#### Planning for retirement

Financial, health, social life, and psychological planning behaviors for retirement were measured using the four subscales of the retirement planning scale (Law et al., 2006). The subscales had satisfactory internal consistency (Cronbach’s alpha = 0.872) in a local study (Liu et al., 2021). The financial planning subscale comprises five items; the health planning and social life planning subscales both contain four items; and the psychological planning subscale has seven items. The participants responded 1 (*yes*) or 0 (*no*) to indicate whether they had engaged in the planning activity. The score ranges of the four subscales were thus 0–5, 0–4, 0–4, and 0–7, respectively, with a higher score indicating a greater level of engagement in planning activities.

#### Sociodemographic characteristics

Age, gender, educational attainment, self-rated socioeconomic status, and health status data were collected. Education was categorized into (a) primary or no formal education and (b) secondary or higher education. Socioeconomic status was categorized into (a) lower class or lower-middle class and (b) middle class or higher. Health status was measured using a single item inquiring into self-rated degree of general health; this item was scored using a 5-point scale ranging from *poor* to *excellent* (Ware & Sherbourne, 1992). The total score ranged from 1 to 5, with a higher score indicating better health.

### Data Analysis

**Figure 1.**
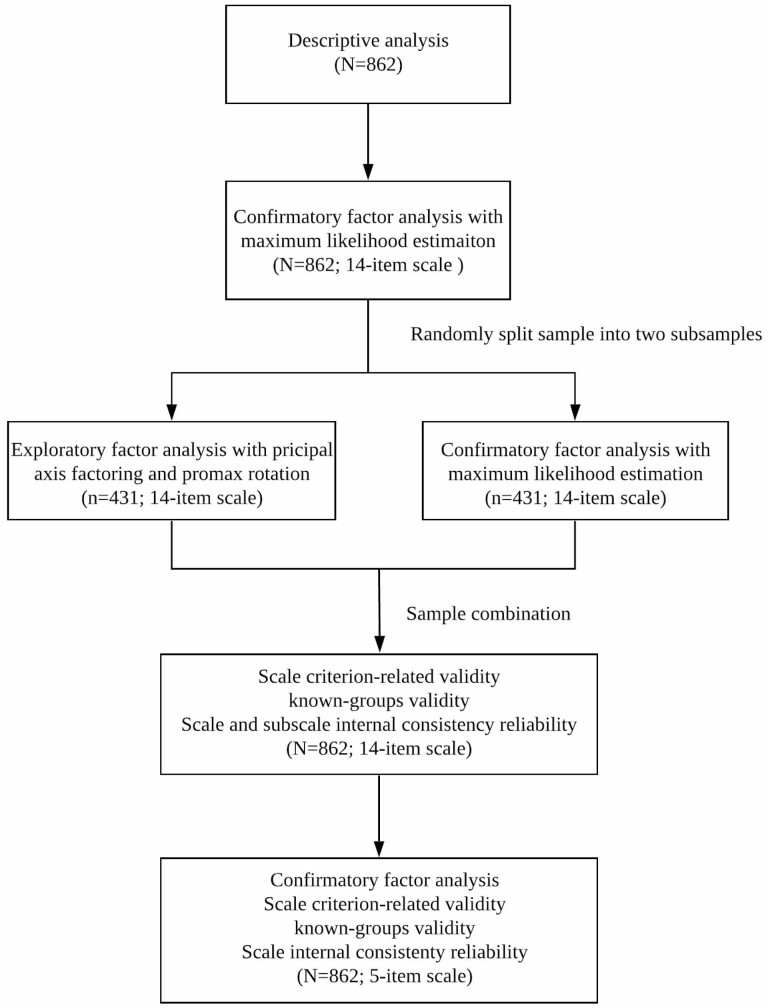
Flowchart of data analysis process.

All data analyses were performed using SPSS 24 and Amos 23. Figure 1 presents a flowchart of the data analysis process. Descriptive analyses were conducted to obtain the mean and *SD* or frequencies and percentages of key variables. Structural validity was assessed by factor analysis. An initial round of confirmatory factor analysis (CFA) was conducted with the whole sample (*N* = 862) to examine whether the data fit the theoretical five-factor model. A relative chi-square value (CMIN/df) less than 5 (Schumacker & Lomax, 2004), a goodness of fit index (GFI) higher than 0.9 (Byrne, 1994), a comparative fit index (CFI) higher than 0.93 (Byrne, 1994), and a root-mean-square error of approximation (RMSEA) value lower than 0.08 (Browne & Cudeck, 1993) were set as the criteria for model acceptability.

The model fit of the five-factor model was unsatisfactory; thus, exploratory factor analysis (EFA) was performed using principal axis factoring with promax rotation on a random half (*n* = 431) of the sample (Schmitt, 2011). A new factor structure that was meaningful in explaining the unique care planning pattern under Chinese culture was identified. CFA was then performed on the other half of the sample (*n* = 431) to validate the newly identified factor structure.

After the factor structure had been confirmed, criterion-related validity was examined by testing correlations between the PFCN-14 and participants’ scores for financial, health, social life, and psychological planning for retirement. Known-groups validity was tested by comparing, through independent sample *t* tests, the scores of participants from the following known groups: women versus men, individuals with higher versus lower levels of education, and individuals with higher versus lower self-perceived socioeconomic status. The internal consistency of the 14-item scale was examined using Cronbach’s alpha from the whole sample (*N* = 862).

The factor structure of the 5-item version of the PFCN scale was further examined. During the scale development process, Sörensen and colleagues (2017) conducted a principal component analysis with 12 items from the original 15-item scale, excluding the three items from the avoidance domain. This approach made it possible to force one component and choose one item from each subdomain with the highest factor loading. Therefore, to test the structural validity of the 5-item version in the current study, CFA was performed with four items forming one component, excluding the item from the avoidance domain. The criterion-related validity, known-groups validity, and internal consistency of PFCN-5 were tested in similar ways as PFCN-14.

## Results

Table 1 presents the descriptive statistics of the entire sample and the subsamples that were randomly selected to conduct EFA and CFA. For the entire sample, the average age was 72.544 years and 56.1% of the participants were men. About 50.5% of the participants received secondary or higher education, and 66.1% of the participants rated their socioeconomic status as lower or lower-middle class. The mean score for self-rated health status was 2.612 out of 5, indicating an average level of health. No significant differences between EFA and CFA subsamples in terms of the key variables were recorded.

**Table 1.** Characteristics of the Total Sample, EFA Subsample, and CFA Subsample

Demographics	Total sample (*N* = 862)	EFA subsample (*n* = 431)	CFA subsample (*n* = 431)
Age: mean (*SD*)	72.544 (8.299)	72.483 (8.422)	72.606 (8.184)
Age: *N* (%)			
60–64	162 (18.8)	83 (19.3)	79 (18.3)
65–74	374 (43.4)	187 (43.4)	187 (43.3)
75–84	238 (27.6)	115 (26.7)	123 (28.5)
85 and above	88 (10.2)	46 (10.7)	42 (9.7)
Gender: *N* (%)			
Men	484 (56.1)	257 (59.6)	227 (52.7)
Women	378 (43.9)	174 (40.4)	204 (47.3)
Educational level: *N* (%)			
No education or primary education	426 (49.4)	202 (46.9)	224 (52.0)
Secondary or higher education	435 (50.5)	229 (53.1)	206 (47.8)
Missing	1 (0.1)		1 (0.2)
Self-rated socioeconomic status: *N* (%)			
Lower or lower-middle class	570 (66.1)	287 (66.6)	283 (65.7)
Middle class or higher	271 (31.4)	131 (30.4)	140 (32.5)
Missing	21 (2.4)	13 (3.0)	8 (1.9)
Self-rated health status: mean (*SD*)	2.612 (0.943)	2.640 (0.904)	2.584 (0.981)

*Notes*: CFA = confirmatory factor analysis; EFA = exploratory factor analysis; *SD* = standard deviation.

The results of an initial round of CFA demonstrated that the five-factor model was inadequate for the current sample (CMIN/df = 7.517, CFI = 0.928, GFI = 0.917, RMSEA = 0.087). EFA was then performed on a random half of the sample (*n* = 431). The Kaiser–Meyer–Olkin value was 0.860, and Bartlett’s test of sphericity attained statistical significance (*p* < .001), indicating that the sample met the criteria for factor analysis (Hair et al., 2010). As shown in Table 2, the rotated component matrix yielded four factors with an eigenvalue higher than 1; these factors explained 68.9% of the total variance. All items had single dominant factor loadings higher than 0.4. Factor 1 (Items 4–6; 39.1% of variance explained) measured avoidance, Factor 2 (Items 9 and 12–14; 12.8% of variance explained) measured concrete planning, Factor 3 (Items 1–3 and 8; 9.2% of variance explained) measured awareness, and Factor 4 (Items 7, 10, and 11; 7.9% of variance explained) measured decision making. The communalities of all variables were higher than 0.35, except for Item 9 (0.272), indicating that the items shared some common variance (Tabachnick & Fidell, 2007).

**Table 2.** Analysis of the 14-Item Preparation for Future Care Needs Scale

	*N* = 862			PAF – loadings^a^*n* = 431				
Item	Mean	*SD*	Corrected item-total correlation	Communalities	1	2	3	4
Factor 1: Avoidance of care planning (range [3–15]; VE: 39.1%)	8.704	3.947						
5. I don’t like to think about the risk of needing help or care in the future.	2.817	1.400	0.592	0.910	0.954			
4. I try not to think about things like future loss of independence.	2.901	1.400	0.618	0.848	0.908			
6. I avoid negative topics like future dependence.	2.986	1.421	0.551	0.671	0.816			
Factor 2: Concrete planning (range [4–20]; VE: 12.8%)	8.639	4.005						
14. I have identified how I want to be cared for and taken concrete steps to ensure that option is available.	2.046	1.281	0.535	0.569		0.873		
13. I have explained to someone close to me what my care preferences are.	2.292	1.398	0.595	0.519		0.677		
12. If I ever need help or care, I can choose between several options that I have considered in some depth.	2.606	1.368	0.717	0.665		0.644		
9. I have gathered information about options for care by talking to health care professionals (doctors, nurses, home health care agencies).	1.695	1.094	0.381	0.272		0.433		
Factor 3: Awareness (range [4–20]; VE: 9.2%)	12.056	4.034						
3. Talking to other people has made me think about whether I might need help or care in the future.	2.835	1.354	0.537	0.464			0.715	
2. I pay attention to information in the media on the risks of needing help or care in old age.	3.258	1.316	0.512	0.396			0.666	
1. I pay close attention to how my physical and mental capabilities are changing to assess whether I may soon need help or care.	3.431	1.320	0.490	0.357			0.555	
8. I have gathered information about options for care by talking to friends and/or relatives.	2.531	1.352	0.627	0.524			0.470	
Factor 4: Decision making (range [3–15]; VE: 7.9%)	9.684	3.421						
10. I know what options for care I don’t want.	3.498	1.340	0.492	0.694				0.922
11. I know my general preferences for care in the future even though I am not sure how I will get what I want.	3.235	1.316	0.625	0.654				0.745
7. I have compared different options for obtaining help or care in the future.	2.951	1.412	0.635	0.502				0.404
Total (range [14, 70]; VE = 68.9%)	39.083	12.024						
Cronbach’s alpha (*N* = 862): 0.889					0.928	0.779	0.749	0.792

*Notes*: PAF = principal axis factoring; *SD* = standard deviation; VE = variance explained.

^a^Kaiser–Meyer–Olkin measure of sampling adequacy = 0.860.

*p* Value of Bartlett’s test of sphericity < .001.

**Figure 2.**
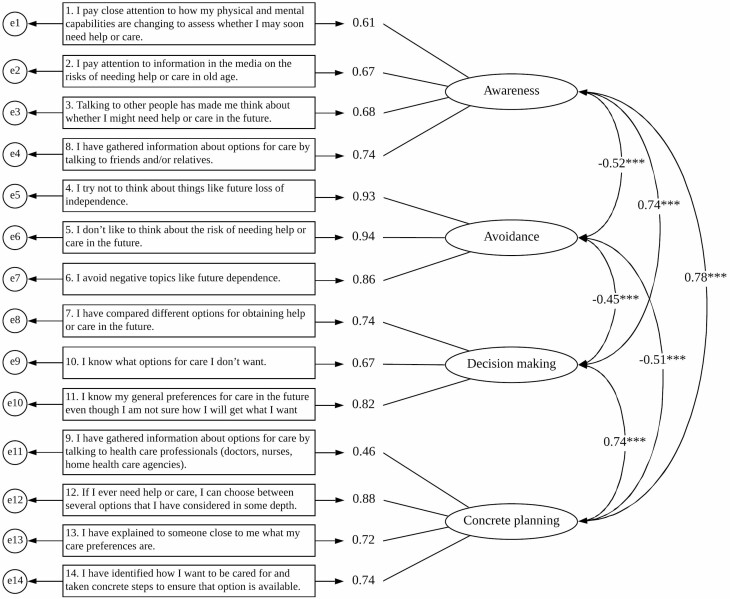
Results of confirmatory factor analysis for 14-item Preparation for Future Care Needs Scale (*N* = 431). *Note*: ***p < .001.

Another round of CFA was performed by using the other random half of the sample (*n* = 431). The standardized parameters, path diagrams, and factor loadings are presented in Figure 2. All factor loadings exceeded 0.6 except for that of Item 9 (0.46). The model exhibited acceptable fit (CMIN/df = 2.767, CFI = 0.960, GFI = 0.943, RMSEA = 0.064). Moreover, the awareness, decision making, and concrete planning domains were significantly and positively correlated with each other; the avoidance domain was negatively correlated with the other three domains.

As presented in Table 2, the mean (*SD*) of the PFCN-14 was 39.083 (12.024), with possible scores ranging from 14 to 70. The mean (*SD*) values of Factors 1 to 4 were 8.704 (3.947), 8.639 (4.005), 12.056 (4.034), and 9.684 (3.421), respectively. The possible scores of Factors 1 and 4 ranged from 3 to 15, whereas those of Factors 2 and 3 range from 4 to 20. The corrected item-total correlations ranged from 0.381 to 0.717, indicating satisfactory levels of correlation (Streiner & Norman, 2003). The Cronbach’s alpha for the scale was 0.889. The subscale Cronbach’s alphas for Factors 1 to 4 were 0.928, 0.779, 0.749, and 0.792, respectively. These results indicated that the scale had satisfactory internal consistency (Hair et al., 2010).

The criterion-related validity of the PFCN-14 was demonstrated by the positive correlations of PFCN-14 total score with financial planning (*r* = 0.315, *p* < .001), health planning (*r* = 0.303, *p* < .001), social life planning (*r* = 0.292, *p* < .001), and psychological planning (*r* = 0.290, *p* < .001) behaviors. As presented in Table 3, known-groups validity was established because the scores were significantly higher for female participants, participants with higher educational attainment, and those with higher socioeconomic status than for male participants and those with lower education and socioeconomic status, respectively.

**Table 3.** Known-Groups Validity of PFCN-14 and PFCN-5 (*N* = 862)

Group	Mean scores of PFCN-14	Mean scores of PFCN-5
Gender		
Male	37.372	12.373
Female	41.274	14.180
*t* Value	−4.788***	−5.369***
Educational level		
No formal education or primary education	36.083	12.016
Secondary education or above	42.033	14.298
*t* Value	−7.486***	−6.896***
Self-perceived socioeconomic status		
Lower class or lower-middle class	37.363	12.412
Middle class or higher	42.780	14.778
*t* Value	−6.232***	−6.581***

*Notes*: PFCN-5 = 5-item Preparation for Future Care Needs Scale PFCN-14 = 14-item Preparation for Future Care Needs Scale.

*t* Values were calculated with independent sample *t* tests; ***p < .001.

**Figure 3.**
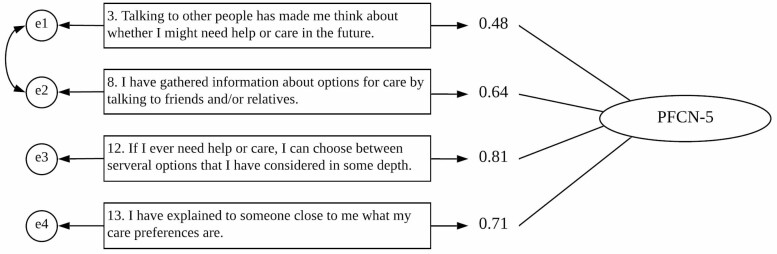
Results of confirmatory factor analysis for 5-item Preparation for Future Care Needs Scale (*N* = 862).

To validate the PFCN-5, CFA was performed with four items (excluding Item 4) on the entire sample (*N* = 862). Covariance between the errors of Items 3 and 8 was added within the factor because the two items appeared to be correlated and the correlation was theoretically meaningful. Figure 3 presents the results estimated using the standardized parameters with path diagrams and factor loadings. The satisfactory model fit confirmed the structural validity of the scale (CMIN/df = 3.551, CFI = 0.997, GFI = 0.998, and RMSEA = 0.054).

The criterion-related validity of the PFCN-5 was demonstrated by the positive correlations of PFCN-5 total score with financial planning (*r* = 0.299, *p* < .001), health planning (*r* = 0.287, *p* < .001), social life planning (*r* = 0.295, *p* < .001), and psychological planning (*r* = 0.286, *p* < .001) behaviors. As presented in Table 3, the known-groups validity of the scale was established by the significantly higher scores for female participants and those with higher educational attainment and socioeconomic status compared with male participants and those with lower education and socioeconomic status, respectively. The Cronbach’s alpha for the scale was 0.774.

## Discussion and Implications

This study is the first to validate the Chinese versions of two short forms of the PFCN scale to assess aging adults’ preparation processes for their future care needs. The Chinese version PFCN-14 yielded a four-factor solution comprising awareness, avoidance, decision making, and concrete planning domains. This factorial structure captured three of the four main steps of the care planning process as suggested by the original PFCN model, but it also reflected a unique pattern of care planning in Chinese older adults. Influenced by the cultural value of family care and by the limited formal care options available in Hong Kong, the “information-gathering” step was skipped by Chinese older adults. The items intended to measure the information-gathering domain loaded onto the other three domains. This factor pattern reflected Chinese older adults’ passive information-gathering behaviors and their reliance on family members in care planning. The identification and validation of the four-factor model contributed to our understanding of the unique care planning behaviors of those with a Chinese cultural background.

### Information gathering: A Missing Factor in PFCN-14

Interpreting the item-level differences in the Chinese PFCN scale, an item from the original information-gathering subscale (“I have gathered information about options for care by talking to friends and/or relatives”) loaded onto the awareness domain. This result reflected unique cultural influences on the care planning process of Chinese older adults. The family has long been considered the primary source of care for older people in Chinese societies; thus, for each older adult, future care expectations and arrangements are likely to be related to family dynamics and function (Bai, 2019a, 2019b). Therefore, older Chinese people may be reluctant to discuss their future care, which is a private issue, with friends or purposefully gather information and advice. From their perspective, talking to relatives or friends is a passive process that contributes to the awareness that they may need care one day (Yap et al., 2018) rather than an active process of information gathering. Moreover, with an awareness of future care needs, older people may become more attentive to relevant information when talking with their friends, but these discussions are not motivated by intentional information seeking.

Another item in the information-gathering domain (“I have compared different options for obtaining help or care in the future”) loaded onto the decision-making domain. This finding indicated that for Chinese older adults, evaluations of different care options were not necessarily based on active information gathering; instead, the older adults may weigh care options according to their strong preferences for specific arrangements and to available resources. For example, due to traditional cultural values related to family care, institutional care may be perceived as unacceptable or disrespectable (Song et al., 2018; Bai et al., 2020) regardless of whether the person has detailed information on this option. Similarly, even without collecting sufficient information, some older people are aware of the infeasibility of hiring domestic workers as caregivers considering their disadvantaged financial situation (Bai et al., 2020; Ochiai, 2011). Consequently, in the older Chinese population, comparisons of care choices are related more to decision making than to information gathering.

The last item in the information-gathering domain (“I have gathered information about options for care by talking to health care professionals”) loaded onto the concrete planning factor. In contrast to other medical treatment decisions that require a consultation with health care professionals, care arrangements for older Chinese people are largely dependent on personal preferences and resources. Therefore, suggestions from care professionals may not be perceived as crucial in decision making. Instead of seeking information, older Chinese people may only talk to professionals when they have questions regarding more concrete care plans or when they have medical considerations that require complex care planning.

### Levels of Preparation in Different Care Planning Steps

Compared with the participants of the study conducted by Sörensen et al. (2017) in the United States, Chinese older adults appeared less likely to engage in care preparation activities (mean item score: 3.09 vs 2.79) and demonstrated lower levels of planning in all subdomains. Considering that the participants in the U.S. study were aged 65 years and older and the participants in this study were aged 60 years and older, we calculated the mean item score of our participants aged 65 years and older to ensure the comparability of the studies. The mean item score for our participants aged 65 and older was 2.74, lower than that of the U.S. sample. The difference may be due in part to the higher percentage of well-educated participants in the U.S. study. Moreover, sociocultural factors such as the expected reliance on family care may discourage Chinese older adults’ efforts in care planning, and less availability of formal care options is likely to result in less consideration of these sources, although traditional values affect the care preferences of older adults (Pinquart et al., 2018).

Chinese older adults had moderate levels of both awareness of and avoidance of future care needs. The mean scores for specific items indicated that the participants tended to regard future care as a negative topic and avoided thinking about it (Item 6). As mentioned, perceiving future care needs as a death-related topic, Chinese older adults may avoid thinking about and planning for future care. Alternatively, older adults in Hong Kong may be beginning to realize that their most preferred care source (i.e., family care) is becoming less available and reliable (Bai, 2019b; Bai et al., 2020), and other acceptable care options (e.g., hiring domestic workers) are only feasible for those who are financially advantaged. Therefore, limited care options and the anticipation of receiving undesirable care may prompt negative emotional arousal that distract from responding to potential stressors (i.e., future care needs) and interfere with the ability to cope (Aspinwall & Taylor, 1997). Additionally, this avoidance tendency may be due to a lack of information support, planning skills, and other resources that are essential during the proactive coping stage (Aspinwall & Taylor, 1997).

Unlike in Sörensen et al.’s (2017) study, the avoidance domain was found negatively related to the other domains in current study. This difference suggested that avoidance may have more severe negative effects on the proactive care planning behaviors of Chinese older adults than on those of U.S. older adults. Service practitioners should develop public education programs to disassociate preparing for future care needs from death-related topics, for example, by encouraging older adults to proactively arrange for a variety of future needs, including needs for socializing, meaningful activity, and care. The positive outcomes of a program in Iowa suggested that this approach is promising (Lee et al., 2019; Sörensen et al., 2021). Furthermore, eldercare information should be more accessible to its target population, and training programs should be provided for older adults and their family members to develop care planning skills.

The decision-making domain yielded the highest mean item score among all subdomains, reflecting that older people in Hong Kong had clear care preferences even if they felt uncertain about whether they could achieve them. Item 10 (“I know what options for care I don’t want.”) and Item 11 (“I know my general preferences for care in the future even though I am not sure how I will get what I want.”) in this domain yielded the highest scores. This finding is consistent with a previous study, which found that some older people in Hong Kong regarded institutional care as a last resort due to the seemingly poor quality of some care facilities (Bai et al., 2020). Moreover, although many people had preferences for future care (e.g., looking forward to hiring a domestic worker), they were not confident in their ability to achieve them due to financial limitations (Bai et al., 2020).

Concrete planning had the lowest rating out of all the subdomains. Among the four items measuring this domain, Item 9 (“I have gathered information about options for care by talking to health care professionals”) yielded the lowest score. In Hong Kong, future care planning is still a new concept that is unfamiliar to many older people and their family members, so it may not be commonplace to consult health care professionals for advice. Furthermore, as revealed by previous studies conducted in the United States, overconfidence in personal health status (Girling & Morgan, 2014), perceived uselessness of care planning (Sörensen & Pinquart, 2000b), and limited access to resources (Pinquart et al., 2003) may also result in low levels of engagement in concrete care planning. To develop tailored policies and services to improve care planning, future studies are needed to thoroughly investigate the barriers faced by Hong Kong older adults.

Lastly, higher levels of care planning were detected among participants of female gender and with higher educational attainment and self-perceived socioeconomic status, which is consistent with findings in previous studies (He & Chou, 2019; Kawakami et al., 2021; Song, 2016). Policymakers and service providers should promote public education emphasizing the importance and benefits of care preparation and targeting certain groups of older adults; additionally, support services and policies should be developed for vulnerable older adults to better prepare them for their future care needs.

### Applications of the PFCN-14 and PFCN-5

The Chinese version of the PFCN-14 can serve as a tool for evaluating older adults’ processes of planning for future care needs in Hong Kong. This scale can be employed by social workers to identify older people inadequately prepared for future care needs, determine which planning step an older adult has not yet completed, and inform the development of targeted interventions to assist with care planning. The PFCN-5, by contrast, enables a more general evaluation of older people’s preparation progress and can be used to start a conversation about care preferences, care planning attitude, and actions (Sörensen et al., 2017).

### Limitations

Several limitations of the current study should be acknowledged. First, the use of purposive sampling may have limited the generalizability of the findings. Second, test–retest reliability was not examined to evaluate the stability of the responses, and the convergent validity of the scale was not examined because of the limitation of similar measures of the construct. Finally, considering cultural sensitivity, the scales validated in the current study are only recommended for use in Chinese societies or in societies with similar cultural norms. Future studies may validate the two scales in other societies.

### Conclusion

The current study is the first to validate Chinese versions of the two short forms of the PFCN scale and examine the processes and extent to which Chinese older adults in Hong Kong engage in planning for their future care. Statistical analyses indicated that the internal consistency, structural validity, criterion-related validity, and known-groups validity of the scales were satisfactory. The results in the current study shed new light on the measurement of PFCN and indicated the importance of cultural modification when applying the original PFCN scale. Because the entire factor structure was not culturally invariant and the process of care planning was influenced by cultural differences, we should pay attention to the conceptualization of the care planning process under different cultural backgrounds.

## Funding

This work was supported by the General Research Fund from the Research Grants Council of the Hong Kong Special Administrative Region, China (grant number: 15603818); and The Hong Kong Polytechnic University (grant number: P0008732, P0033585).

## Conflict of Interest

None declared.
